# 5-Amino-1-phenyl-1*H*-pyrazole-4-carboxylic acid

**DOI:** 10.1107/S1600536808018394

**Published:** 2008-06-21

**Authors:** Muhammad Zia-ur-Rehman, Mark R. J. Elsegood, Nosheen Akbar, Rahman Shah Zaib Saleem

**Affiliations:** a Applied Chemistry Research Centre, PCSIR Laboratories Complex, Lahore 54600, Pakistan; b Chemistry Department, Loughborough University, Loughborough LE11 3TU, England; c Centre for High Energy Physics, University of the Punjab, Lahore 54590, Pakistan; d Department of Chemistry, Michigan State University, East Lansing, Michigan 48824, USA

## Abstract

In the mol­ecule of the title compound, C_10_H_9_N_3_O_2_, the pyrazole ring is approximately coplanar with the amino and carboxyl groups. The phenyl group is twisted by 48.13 (3)° relative to this plane. An intra­molecular N—H⋯O hydrogen bond stabilizes the planar conformation of the mol­ecule. The mol­ecules are linked into two-dimensional sheets by two strong inter­molecular N—H⋯N and O—H⋯O hydrogen bonds. The latter forms the classic carboxylic acid dimer motif.

## Related literature

For related literature, see: Baroni & Kovyrzina (1961 ▶); Baraldi *et al.* (1998 ▶); Bruno *et al.* (1990 ▶); Chen & Li (1998 ▶); Cottineau *et al.* (2002 ▶); Dardari *et al.* (2006 ▶); Jin *et al.* (2004 ▶); Li *et al.* (2006 ▶); Londershausen (1996 ▶); Mishra *et al.* (1998 ▶); Neunhoeffer *et al.* (1959 ▶); Siddiqui *et al.* (2007 ▶); Smith *et al.* (2001 ▶); Zhong *et al.* (2006 ▶); Zia-ur-Rehman *et al.* (2005 ▶, 2006 ▶).
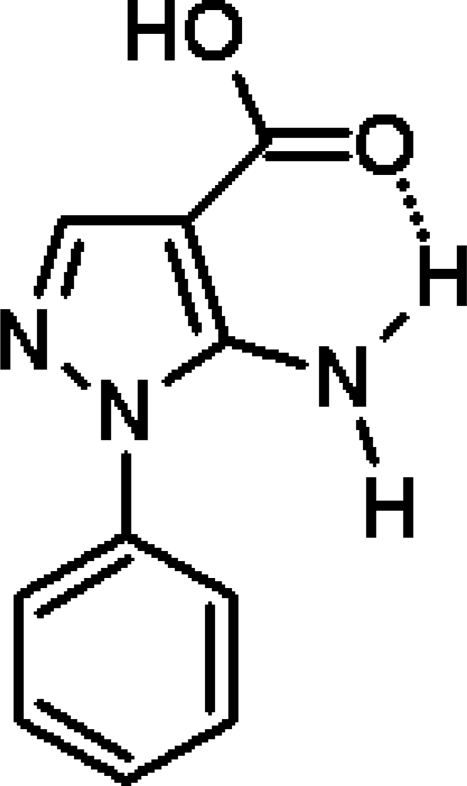


## Experimental

### 

#### Crystal data

C_10_H_9_N_3_O_2_
*M* *_r_* = 203.20
Monoclinic, 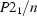

*a* = 3.7937 (5) Å
*b* = 21.613 (3) Å
*c* = 11.1580 (16) Å
β = 92.170 (2)°
*V* = 914.2 (2) Å^3^
*Z* = 4
Mo *K*α radiation
μ = 0.11 mm^−1^
*T* = 150 (2) K
0.28 × 0.10 × 0.07 mm

#### Data collection

Bruker APEXII CCD diffractometer
Absorption correction: multi-scan (*SADABS*; Sheldrick, 2007 ▶) *T*_min_ = 0.971, *T*_max_ = 0.993
10482 measured reflections
2800 independent reflections
1967 reflections with *I* > 2σ(*I*)
*R*_int_ = 0.034

#### Refinement

*R*[*F* ^2^ > 2σ(*F* ^2^)] = 0.043
*wR*(*F* ^2^) = 0.116
*S* = 1.02
2800 reflections
145 parameters
H atoms treated by a mixture of independent and constrained refinement
Δρ_max_ = 0.34 e Å^−3^
Δρ_min_ = −0.27 e Å^−3^

### 

Data collection: *APEX2* (Bruker, 2006 ▶); cell refinement: *SAINT* (Bruker, 2006 ▶); data reduction: *SAINT*; program(s) used to solve structure: *SHELXS97* (Sheldrick, 2008 ▶); program(s) used to refine structure: *SHELXL97* (Sheldrick, 2008 ▶); molecular graphics: *SHELXTL* (Sheldrick, 2008 ▶); software used to prepare material for publication: *SHELXTL* and local programs.

## Supplementary Material

Crystal structure: contains datablocks I, global. DOI: 10.1107/S1600536808018394/bt2722sup1.cif

Structure factors: contains datablocks I. DOI: 10.1107/S1600536808018394/bt2722Isup2.hkl

Additional supplementary materials:  crystallographic information; 3D view; checkCIF report

## Figures and Tables

**Table 1:** Hydrogen-bond geometry (Å, °)

*D*—H⋯*A*	*D*—H	H⋯*A*	*D*⋯*A*	*D*—H⋯*A*
N4—H4*A*⋯O3	0.903 (18)	2.136 (18)	2.8233 (16)	132.3 (14)
N4—H4*B*⋯N3^i^	0.876 (18)	2.239 (18)	3.0087 (17)	146.5 (15)
O4—H4⋯O3^ii^	0.92 (2)	1.70 (2)	2.6189 (14)	178.4 (19)

Symmetry codes: (i) 
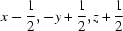
; (ii) 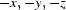.

**Fig. 1.**
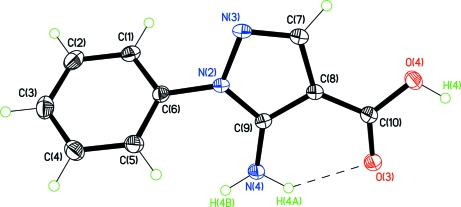
The asymmetric unit of the title compound. Displacement ellipsoids are drawn at the 50% probability level.

**Fig. 2.**
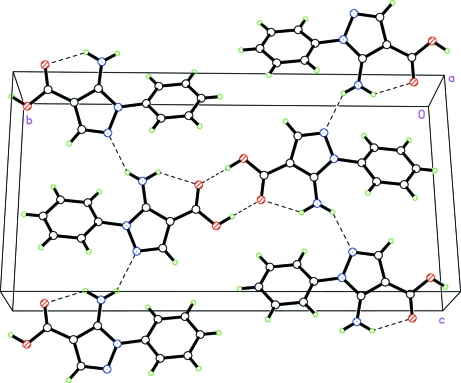
Perspective view of the crystal packing showing hydrogen-bond interactions (dashed lines). H atoms not involved in hydrogen bonding have been omitted for clarity.

## supplementary crystallographic information

### Comment

Pyrazole and its derivatives are known as heterocyclic compounds, having a wide range of biological activities. Some pyrazoles have been reported to possess significant antiarrhythmic and sedative (Bruno *et al.*, 1990), hypoglycemic (Cottineau *et al.*, 2002), antiviral (Baraldi *et al.*, 1998), and pesticidal (Londershausen,1996) activities. Some of their derivatives have also been successfully tested for their antifungal (Chen & Li, 1998), antihistaminic (Mishra *et al.*, 1998) and anti-inflammatory (Smith *et al.*, 2001) activities. In addition, they have also been used as ligands to investigate the structure–activity relationship of the active site of metalloproteins (Dardari *et al.*, 2006) and for the preparation of some commercially important dyestuffs (Baroni & Kovyrzina, 1961; Neunhoeffer *et al.*, 1959).

As part of our ongoing research on the synthesis and biological evaluation of heterocyclic compounds (Zia-ur-Rehman *et al.*, 2005, 2006; Siddiqui *et al.*, 2007), the crystal structure of the title compound, (I), was determined. In (I), the pyrazole ring is approximately co-planar with the amino and carboxylic acid groups. The C—N bond lengths in the pyrazole ring are 1.3146 (18) and 1.3530 (16) Å, which are shorter than a typical C—N single bond length of 1.443 Å, but longer than a typical C—N bond length of 1.269 Å (Jin *et al.*, 2004), indicating electron delocalization. Most of the bond lengths and angles in *N*-phenylpyrazole group are in consistent with those in similar molecules (Li *et al.*, 2006; Zhong *et al.*, 2006). Each molecule exhibits an intramolecular N—H···O hydrogen bond which stabilizes the planar conformation and is linked to an adjacent one through head-to-tail pairs of O—H···O intermolecular interactions giving rise to dimeric motifs typical for carboxylic acids. Neighbouring dimers are further arranged into two-dimensional sheets in the (101) plane through N—H···N interactions (Fig.2).

### Experimental

A mixture of 5-amino-1-phenyl-1*H*-pyrazole-4-carboxylic acid, ethyl ester (2.312 g; 10.0 mmoles), potassium hydroxide (1.12 g; 20 mmoles) and ethanol (25 ml) was refluxed for two hours. The reaction mixture was poured into ice cooled water and acidified with dilute hydrochloric acid to Congo Red. The precipitated solids were collected by filtration, washed and dried. Crystals suitable for single-crystal X-ray diffraction were grown by slow evaporation of solution of the title compound in a mixture of ethanol and water (85:15); m.p. 460 K; yield: 68%.

### Refinement

H atoms bound to C were placed in geometric positions (C—H distance = 0.95 Å) using a riding model. H atoms on N and O had coordinates freely refined. *U*_iso_ values were set to 1.2*U*_eq_ (1.5*U*_eq_ for OH).

### Crystal data

**Table:**

C_10_H_9_N_3_O_2_	*F*_000_ = 424
*M**_r_* = 203.20	*D*_x_ = 1.476 Mg m^−^^3^
Monoclinic, *P*2_1_/*n*	Melting point: 460 K
Hall symbol: -P 2yn	Mo *K*α radiation λ = 0.71073 Å
*a* = 3.7937 (5) Å	Cell parameters from 2299 reflections
*b* = 21.613 (3) Å	θ = 3.4–29.6º
*c* = 11.1580 (16) Å	µ = 0.11 mm^−^^1^
β = 92.170 (2)º	*T* = 150 (2) K
*V* = 914.2 (2) Å^3^	Block, colourless
*Z* = 4	0.28 × 0.10 × 0.07 mm

### Data collection

**Table:**

Bruker APEXII CCD diffractometer	2800 independent reflections
Radiation source: fine-focus sealed tube	1967 reflections with *I* > 2σ(*I*)
Monochromator: graphite	*R*_int_ = 0.034
*T* = 150(2) K	θ_max_ = 30.6º
ω rotation with narrow frames scans	θ_min_ = 1.9º
Absorption correction: multi-scan(SADABS; Sheldrick, 2007)	*h* = −5→5
*T*_min_ = 0.971, *T*_max_ = 0.993	*k* = −30→30
10482 measured reflections	*l* = −15→15

### Refinement

**Table:**

Refinement on *F*^2^	Secondary atom site location: difference Fourier map
Least-squares matrix: full	Hydrogen site location: geom except NH & OH coords freely refined
*R*[*F*^2^ > 2σ(*F*^2^)] = 0.043	H atoms treated by a mixture of independent and constrained refinement
*wR*(*F*^2^) = 0.116	*w* = 1/[σ^2^(*F*_o_^2^) + (0.0521*P*)^2^ + 0.3077*P*] where *P* = (*F*_o_^2^ + 2*F*_c_^2^)/3
*S* = 1.02	(Δ/σ)_max_ < 0.001
2800 reflections	Δρ_max_ = 0.34 e Å^−^^3^
145 parameters	Δρ_min_ = −0.27 e Å^−^^3^
Primary atom site location: structure-invariant direct methods	Extinction correction: none

### Special details

**Table:**

Geometry. All e.s.d.'s (except the e.s.d. in the dihedral angle between two l.s. planes) are estimated using the full covariance matrix. The cell e.s.d.'s are taken into account individually in the estimation of e.s.d.'s in distances, angles and torsion angles; correlations between e.s.d.'s in cell parameters are only used when they are defined by crystal symmetry. An approximate (isotropic) treatment of cell e.s.d.'s is used for estimating e.s.d.'s involving l.s. planes.
Refinement. Refinement of *F*^2^ against ALL reflections. The weighted *R*-factor *wR* and goodness of fit *S* are based on *F*^2^, conventional *R*-factors *R* are based on *F*, with *F* set to zero for negative *F*^2^. The threshold expression of *F*^2^ > σ(*F*^2^) is used only for calculating *R*-factors(gt) *etc*. and is not relevant to the choice of reflections for refinement. *R*-factors based on *F*^2^ are statistically about twice as large as those based on *F*, and *R*- factors based on ALL data will be even larger.

### Fractional atomic coordinates and isotropic or equivalent isotropic displacement parameters (Å^2^)

**Table:**

	*x*	*y*	*z*	*U*_iso_*/*U*_eq_	
C1	0.0569 (4)	0.34024 (6)	−0.20989 (12)	0.0196 (3)	
H1	−0.0345	0.3206	−0.2807	0.023*	
C2	0.0428 (4)	0.40410 (7)	−0.19887 (13)	0.0242 (3)	
H2	−0.0548	0.4284	−0.2628	0.029*	
C3	0.1711 (4)	0.43247 (7)	−0.09461 (14)	0.0262 (3)	
H3	0.1623	0.4762	−0.0873	0.031*	
C4	0.3122 (4)	0.39693 (7)	−0.00110 (13)	0.0235 (3)	
H4C	0.3958	0.4165	0.0707	0.028*	
C5	0.3323 (4)	0.33300 (6)	−0.01161 (12)	0.0196 (3)	
H5	0.4313	0.3087	0.0522	0.024*	
C6	0.2054 (3)	0.30513 (6)	−0.11682 (11)	0.0168 (3)	
N2	0.2312 (3)	0.23989 (5)	−0.13188 (9)	0.0172 (2)	
N3	0.3587 (3)	0.21589 (5)	−0.23810 (10)	0.0205 (3)	
C7	0.3374 (4)	0.15556 (6)	−0.22523 (12)	0.0200 (3)	
H7	0.4084	0.1268	−0.2839	0.024*	
C8	0.1976 (4)	0.13829 (6)	−0.11488 (11)	0.0175 (3)	
C9	0.1300 (3)	0.19438 (6)	−0.05734 (11)	0.0163 (3)	
N4	−0.0209 (3)	0.20277 (6)	0.04849 (10)	0.0221 (3)	
H4A	−0.068 (5)	0.1668 (8)	0.0857 (15)	0.027*	
H4B	−0.028 (5)	0.2377 (8)	0.0881 (16)	0.027*	
C10	0.1212 (4)	0.07860 (6)	−0.06630 (12)	0.0198 (3)	
O3	−0.0099 (3)	0.07239 (4)	0.03332 (9)	0.0247 (2)	
O4	0.1962 (3)	0.03107 (5)	−0.13546 (9)	0.0289 (3)	
H4	0.132 (5)	−0.0050 (10)	−0.0983 (17)	0.043*	

### Atomic displacement parameters (Å^2^)

**Table:**

	*U*^11^	*U*^22^	*U*^33^	*U*^12^	*U*^13^	*U*^23^
C1	0.0207 (7)	0.0229 (7)	0.0153 (6)	0.0004 (5)	0.0039 (5)	0.0023 (5)
C2	0.0263 (7)	0.0229 (7)	0.0239 (7)	0.0054 (6)	0.0068 (6)	0.0071 (5)
C3	0.0298 (8)	0.0172 (6)	0.0324 (8)	−0.0002 (6)	0.0113 (6)	−0.0003 (5)
C4	0.0257 (7)	0.0229 (7)	0.0222 (7)	−0.0043 (5)	0.0052 (5)	−0.0049 (5)
C5	0.0210 (7)	0.0208 (6)	0.0171 (6)	−0.0009 (5)	0.0011 (5)	−0.0005 (5)
C6	0.0183 (6)	0.0163 (6)	0.0161 (6)	−0.0002 (5)	0.0046 (5)	0.0009 (5)
N2	0.0238 (6)	0.0162 (5)	0.0119 (5)	−0.0004 (4)	0.0040 (4)	0.0001 (4)
N3	0.0285 (6)	0.0208 (6)	0.0126 (5)	0.0010 (5)	0.0069 (4)	−0.0007 (4)
C7	0.0266 (7)	0.0192 (6)	0.0144 (6)	0.0006 (5)	0.0042 (5)	−0.0011 (5)
C8	0.0227 (6)	0.0164 (6)	0.0136 (6)	−0.0001 (5)	0.0029 (5)	0.0000 (4)
C9	0.0195 (6)	0.0164 (6)	0.0133 (6)	−0.0004 (5)	0.0013 (5)	0.0009 (4)
N4	0.0348 (7)	0.0168 (5)	0.0153 (5)	−0.0022 (5)	0.0095 (5)	−0.0009 (4)
C10	0.0256 (7)	0.0173 (6)	0.0165 (6)	−0.0001 (5)	0.0034 (5)	−0.0009 (5)
O3	0.0384 (6)	0.0179 (5)	0.0185 (5)	−0.0016 (4)	0.0096 (4)	0.0006 (4)
O4	0.0493 (7)	0.0158 (5)	0.0228 (5)	−0.0021 (5)	0.0157 (5)	−0.0022 (4)

### Geometric parameters (Å, °)

**Table:**

C1—C2	1.387 (2)	N2—N3	1.3968 (15)
C1—C6	1.3883 (18)	N3—C7	1.3146 (18)
C1—H1	0.9500	C7—C8	1.4092 (17)
C2—C3	1.387 (2)	C7—H7	0.9500
C2—H2	0.9500	C8—C9	1.4001 (17)
C3—C4	1.387 (2)	C8—C10	1.4331 (18)
C3—H3	0.9500	C9—N4	1.3438 (16)
C4—C5	1.3891 (19)	N4—H4A	0.903 (18)
C4—H4C	0.9500	N4—H4B	0.876 (18)
C5—C6	1.3891 (18)	C10—O3	1.2423 (16)
C5—H5	0.9500	C10—O4	1.3221 (16)
C6—N2	1.4239 (16)	O4—H4	0.92 (2)
N2—C9	1.3530 (16)		
			
C2—C1—C6	119.58 (13)	C9—N2—C6	128.69 (11)
C2—C1—H1	120.2	N3—N2—C6	119.69 (10)
C6—C1—H1	120.2	C7—N3—N2	104.53 (10)
C3—C2—C1	120.06 (13)	N3—C7—C8	112.64 (12)
C3—C2—H2	120.0	N3—C7—H7	123.7
C1—C2—H2	120.0	C8—C7—H7	123.7
C4—C3—C2	119.97 (13)	C9—C8—C7	104.64 (11)
C4—C3—H3	120.0	C9—C8—C10	124.25 (12)
C2—C3—H3	120.0	C7—C8—C10	131.08 (12)
C3—C4—C5	120.55 (13)	N4—C9—N2	125.61 (12)
C3—C4—H4C	119.7	N4—C9—C8	127.68 (12)
C5—C4—H4C	119.7	N2—C9—C8	106.64 (11)
C4—C5—C6	118.97 (13)	C9—N4—H4A	112.7 (11)
C4—C5—H5	120.5	C9—N4—H4B	125.6 (11)
C6—C5—H5	120.5	H4A—N4—H4B	120.0 (16)
C1—C6—C5	120.86 (13)	O3—C10—O4	122.72 (12)
C1—C6—N2	118.73 (12)	O3—C10—C8	121.96 (12)
C5—C6—N2	120.40 (12)	O4—C10—C8	115.31 (12)
C9—N2—N3	111.54 (10)	C10—O4—H4	109.2 (12)
			
C6—C1—C2—C3	−1.1 (2)	N3—C7—C8—C9	0.05 (16)
C1—C2—C3—C4	−0.3 (2)	N3—C7—C8—C10	178.11 (14)
C2—C3—C4—C5	1.2 (2)	N3—N2—C9—N4	−176.22 (12)
C3—C4—C5—C6	−0.7 (2)	C6—N2—C9—N4	0.3 (2)
C2—C1—C6—C5	1.6 (2)	N3—N2—C9—C8	0.98 (15)
C2—C1—C6—N2	−177.34 (12)	C6—N2—C9—C8	177.55 (13)
C4—C5—C6—C1	−0.7 (2)	C7—C8—C9—N4	176.51 (14)
C4—C5—C6—N2	178.20 (12)	C10—C8—C9—N4	−1.7 (2)
C1—C6—N2—C9	−130.01 (14)	C7—C8—C9—N2	−0.62 (15)
C5—C6—N2—C9	51.1 (2)	C10—C8—C9—N2	−178.85 (13)
C1—C6—N2—N3	46.33 (17)	C9—C8—C10—O3	−0.8 (2)
C5—C6—N2—N3	−132.62 (13)	C7—C8—C10—O3	−178.56 (14)
C9—N2—N3—C7	−0.93 (15)	C9—C8—C10—O4	178.38 (13)
C6—N2—N3—C7	−177.85 (12)	C7—C8—C10—O4	0.7 (2)
N2—N3—C7—C8	0.51 (16)		

### Hydrogen-bond geometry (Å, °)

**Table:**

*D*—H···*A*	*D*—H	H···*A*	*D*···*A*	*D*—H···*A*
N4—H4A···O3	0.903 (18)	2.136 (18)	2.8233 (16)	132.3 (14)
N4—H4B···N3^i^	0.876 (18)	2.239 (18)	3.0087 (17)	146.5 (15)
O4—H4···O3^ii^	0.92 (2)	1.70 (2)	2.6189 (14)	178.4 (19)

Symmetry codes: (i) *x*−1/2, −*y*+1/2, *z*+1/2; (ii) −*x*, −*y*, −*z*.

## References

[bb1] Baraldi, P. G., Manfredini, S., Romagnoli, R., Stevanato, L., Zaid, A. N. & Manservigi, R. (1998). *Nucleosides Nucleotides*, **17**, 2165–2171.

[bb2] Baroni, E. E. & Kovyrzina, K. A. (1961). *Zh. Obshch. Khim.* **31**, 1641–1645.

[bb3] Bruker (2006). *APEX2* and *SAINT* Bruker AXS Inc., Madison, Wisconsin, USA.

[bb4] Bruno, O., Bondavalli, F., Ranise, A., Schenone, P., Losasso, C., Cilenti, L., Matera, C. & Marmo, E. (1990). *Il Farmaco*, **45**, 147–66. 2133992

[bb5] Chen, H. S. & Li, Z. M. (1998). *Chem. J. Chin. Univ.* **19**, 572–576.

[bb6] Cottineau, B., Toto, P., Marot, C., Pipaud, A. & Chenault, J. (2002). *Bioorg. Med. Chem.* **12**, 2105–2108. 10.1016/s0960-894x(02)00380-3 12127514

[bb7] Dardari, Z., Lemrani, M., Sebban, A., Bahloul, A., Hassair, M., Kitane, S., Berrada, M. & Boudouma, M. (2006). *Arch. Pharm***. 339**, 291–298. 10.1002/ardp.200500266 16619283

[bb8] Jin, Z.-M., Li, L., Li, M.-C., Hu, M.-L. & Shen, L. (2004). *Acta Cryst.* C**60**, o642–o643. 10.1107/S0108270104016130 15345843

[bb9] Li, S.-Y., Zhong, P., Hu, M.-L., Luo, Y. & Li, J.-H. (2006). *Acta Cryst.* E**62**, o3821–o3822.

[bb10] Londershausen, M. (1996). *Pestic. Sci.* **48**, 269–274.

[bb11] Mishra, P. D., Wahidullah, S. & Kamat, S. Y. (1998). *Indian J. Chem. Sect. B*, **37**, 199.

[bb12] Neunhoeffer, O., Alsdorf, G. & Ulrich, H. (1959). *Chem. Ber.* **92**, 252–256.

[bb13] Sheldrick, G. M. (2007). *SADABS* University of Göttingen, Germany.

[bb14] Sheldrick, G. M. (2008). *Acta Cryst.* A**64**, 112–122. 10.1107/S0108767307043930 18156677

[bb15] Siddiqui, H. L., Zia-ur-Rehman, M., Ahmad, N., Weaver, G. W. & Lucas, P. D. (2007). *Chem. Pharm. Bull* **55**, 1014–1017. 10.1248/cpb.55.1014 17603192

[bb16] Smith, S. R., Denhardt, G. & Terminelli, C. (2001). *Eur. J. Pharmacol.* **432**, 107–119. 10.1016/s0014-2999(01)01477-7 11734194

[bb17] Zhong, P., Zhang, X.-H., Xiao, H.-P. & Hu, M.-L. (2006). *Acta Cryst.* E**62**, o513–o515.

[bb18] Zia-ur-Rehman, M., Choudary, J. A. & Ahmad, S. (2005). *Bull. Korean Chem. Soc.* **26**, 1771–1175.

[bb19] Zia-ur-Rehman, M., Choudary, J. A., Ahmad, S. & Siddiqui, H. L. (2006). *Chem. Pharm. Bull.* **54**, 1175–1178. 10.1248/cpb.54.1175 16880664

